# Supplementary material for: Colon-Targeted eNAMPT-Specific Peptide Systems for Treatment of DSS-Induced Acute and Chronic Colitis in Mouse
Source: Antioxidants (Basel). 2022 Nov 30;11(12):2376. doi: 10.3390/antiox11122376 (PMC9774280; doi:10.3390/antiox11122376)
Supplement: Supplementary file 1 [file antioxidants-11-02376-s001.zip › antioxidants-1982271-supplementary.pdf]

Supplemental Figures and Tables

A

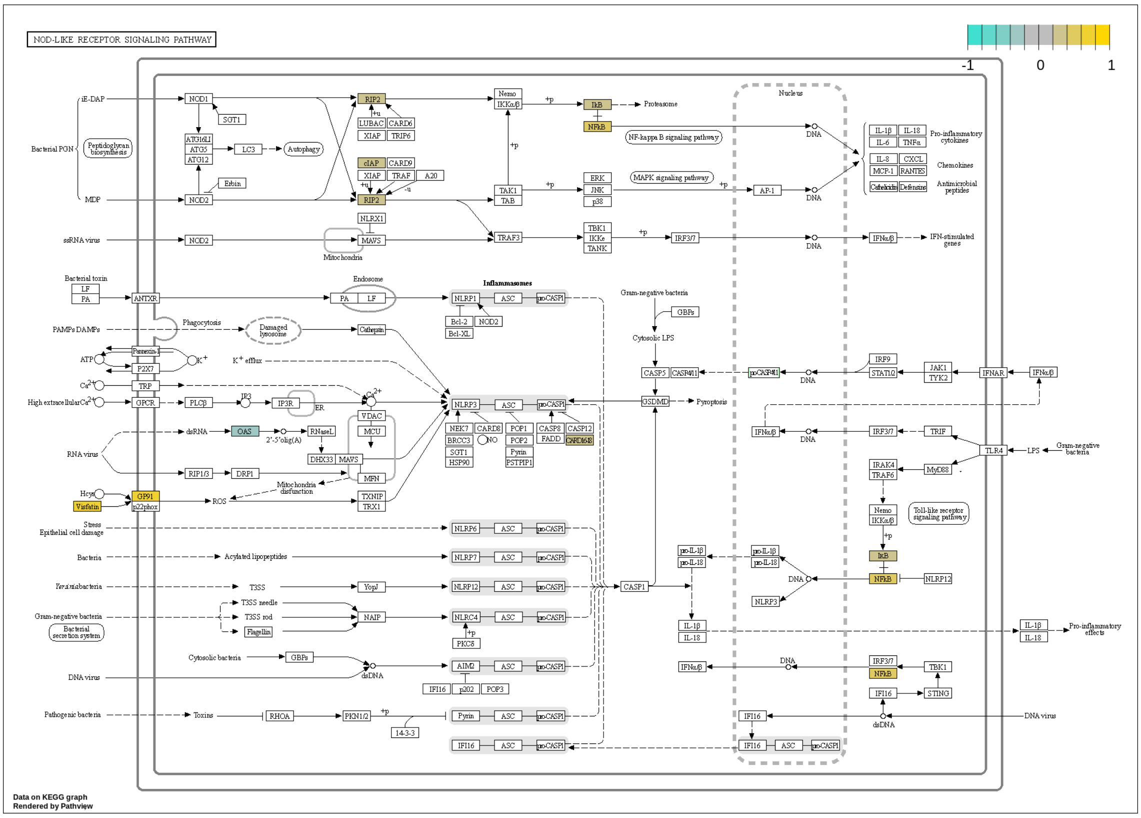

B

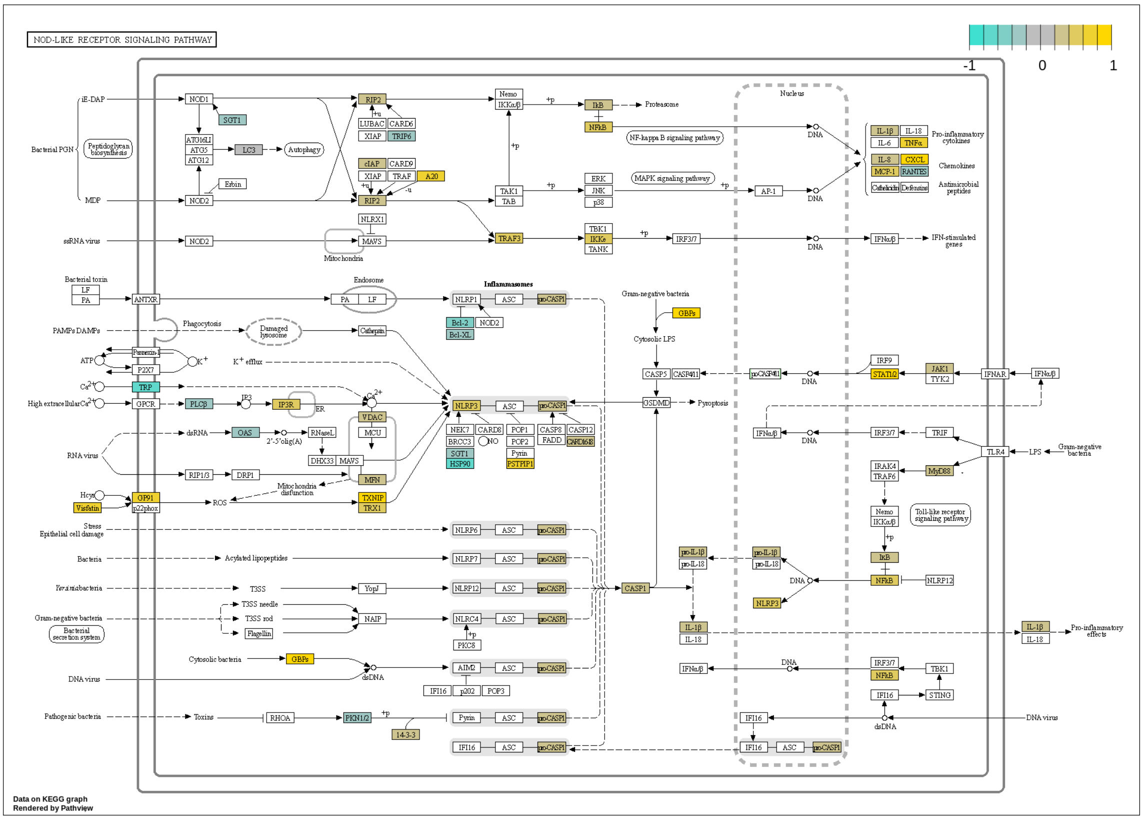

Figure S1. Pathway views of inflamed versus healthy DEG analysis results for macrophage. (**A**) with common DEGs from SCP259 and GSE182270 and (**B**) with DEGs from SCP259 only. With common DEGs, one can identify (1) the NLRP3 inflammasome possibly stimulated by Visfatin(NAMPT) – GP91(CYBB) interaction and (2) NFκB signaling pathway. The activation of the two signaling paths are clearer in (**B**).

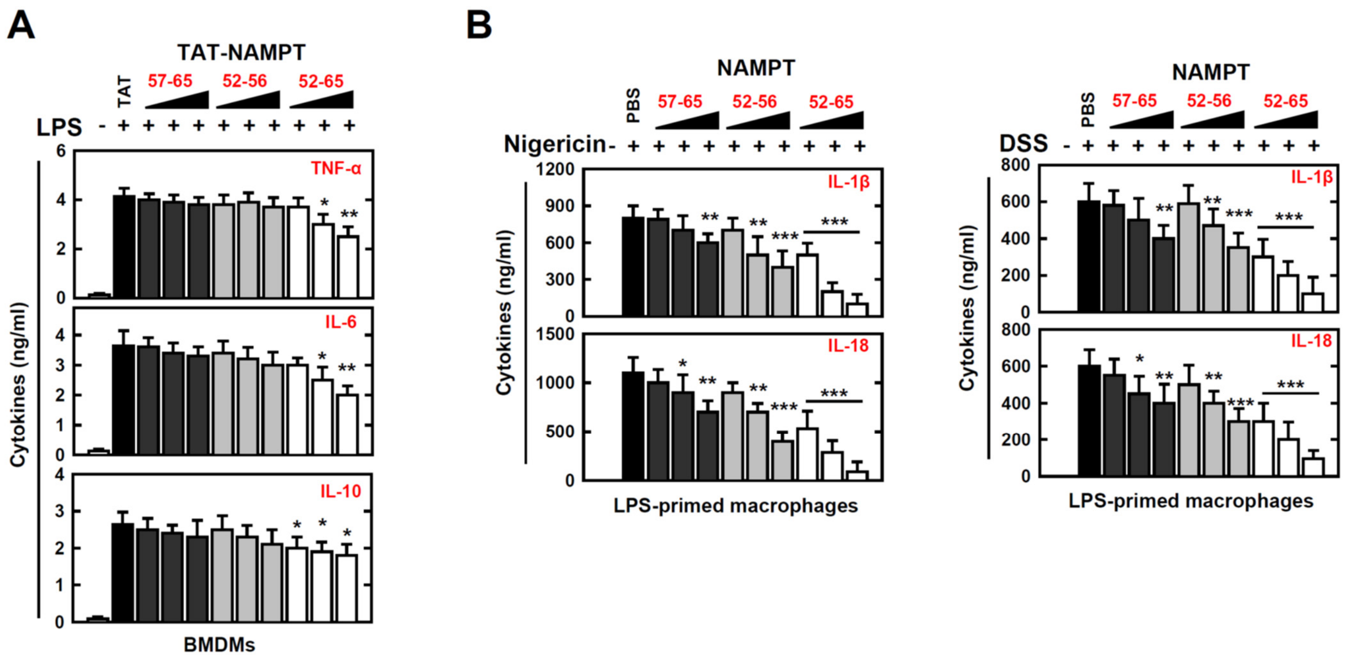

Figure S2. The effects of TAT-NAMPT or NAMPT peptides on inflammation

(**A**) BMDMs were stimulated with LPS (100 ng/mL) for 18 hr, pretreated with rNAMPT for 1 hr. Culture supernatants were harvested and analyzed for cytokine ELISA. (**B**) LPS-primed BMDMs were treated with rNAMPT for 1 h, and then activated with Nigericin for 30 min or DSS for 24 hr. ELISA for IL-1β and IL-18. The data are representative of three independent experiments with similar results. Data shown are the means ± SD of three experiments. Significant differences (*P < 0.05; **P < 0.01; ***P < 0.001) compared with LPS+PBS.

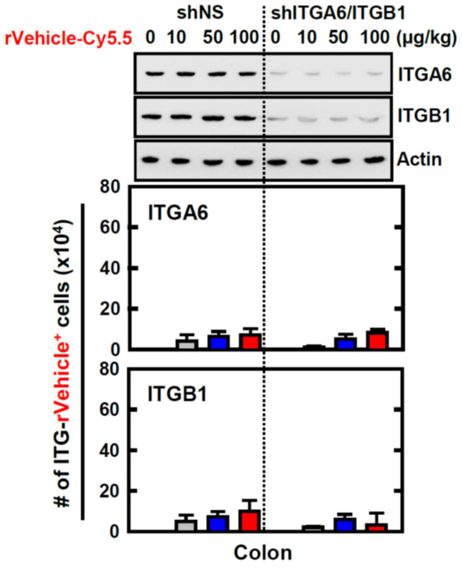

Figure S3. The effects of rVehicle peptides on colon targeting

The experimental conditions followed the protocol outlined in **Fig. 5C.** Colon harvests were used for IB with αITGA6, αITGB1 or αActin and analysis of the number of ITGA6+ or ITGB1+ cells by FACS. The data are representative of three independent experiments with similar results.

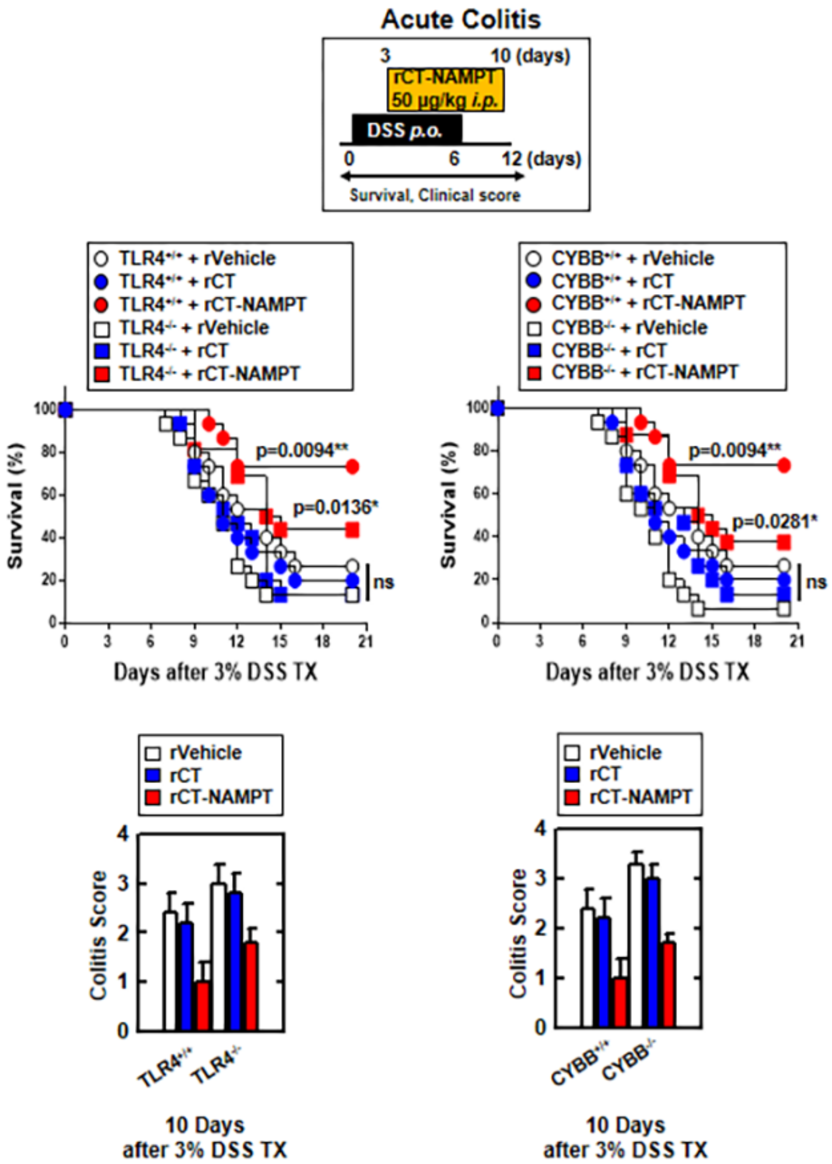

Figure S4. The effects of rCT-NAMPT has a therapeutic effect against acute DSS-induced colitis in TLR4-/- and CYBB-/- mice.

Schematic of the chronic colitis model treated 3% DSS with rCT-NAMPT (50 μg/kg) (upper). The survival of mice was monitored for 12 days; mortality was measured for n = 15 mice per group. Statistical differences compared with the rVehicle-treated mice are indicated (log-rank test). The data are representative of two independent experiments with similar results. Colitis scores were obtained from clinical parameter (weight loss, stool consistency, bleeding) (n = 8, lower).

Figure S5. Full-length images of the blots presented in the Figures.

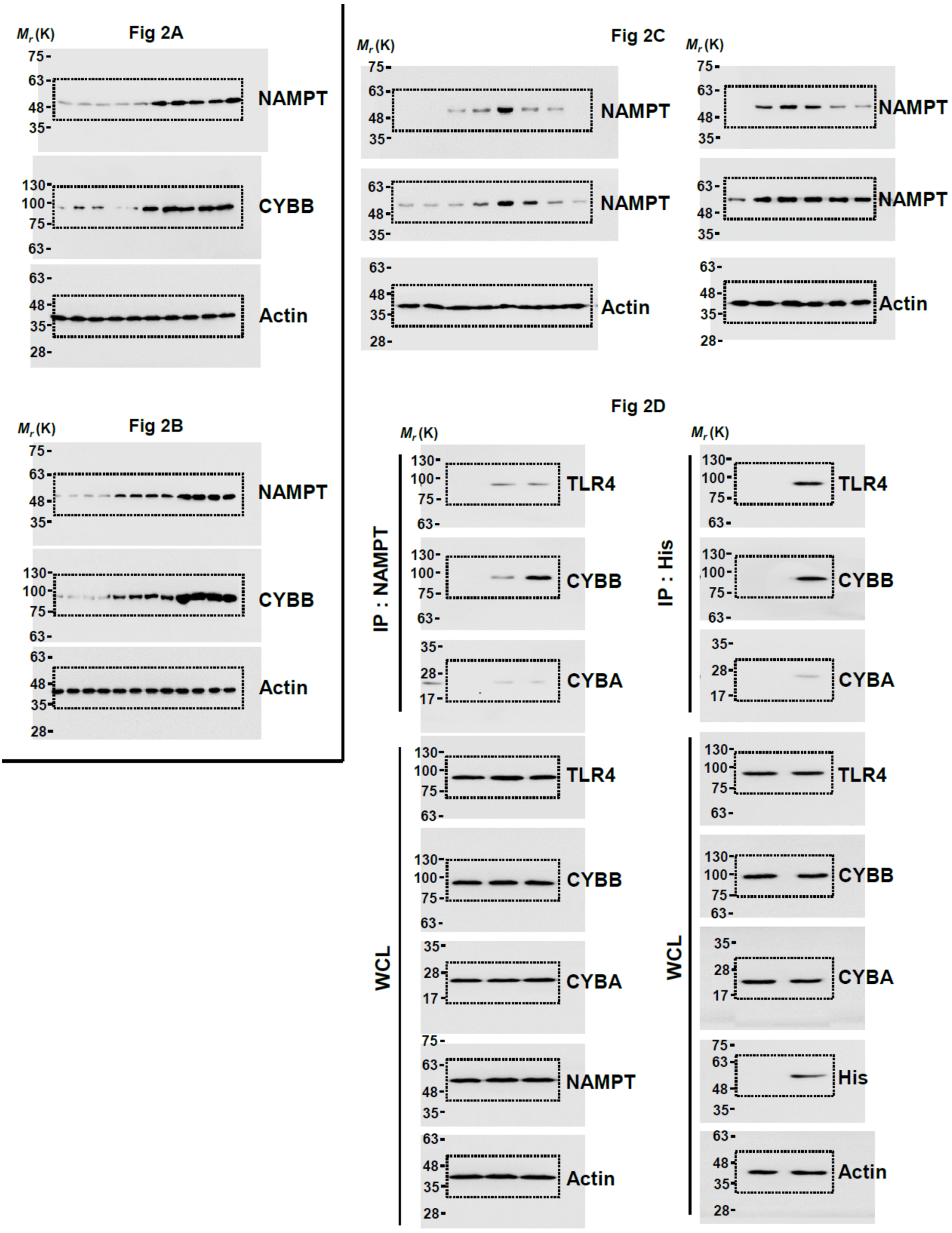

Fig 2E

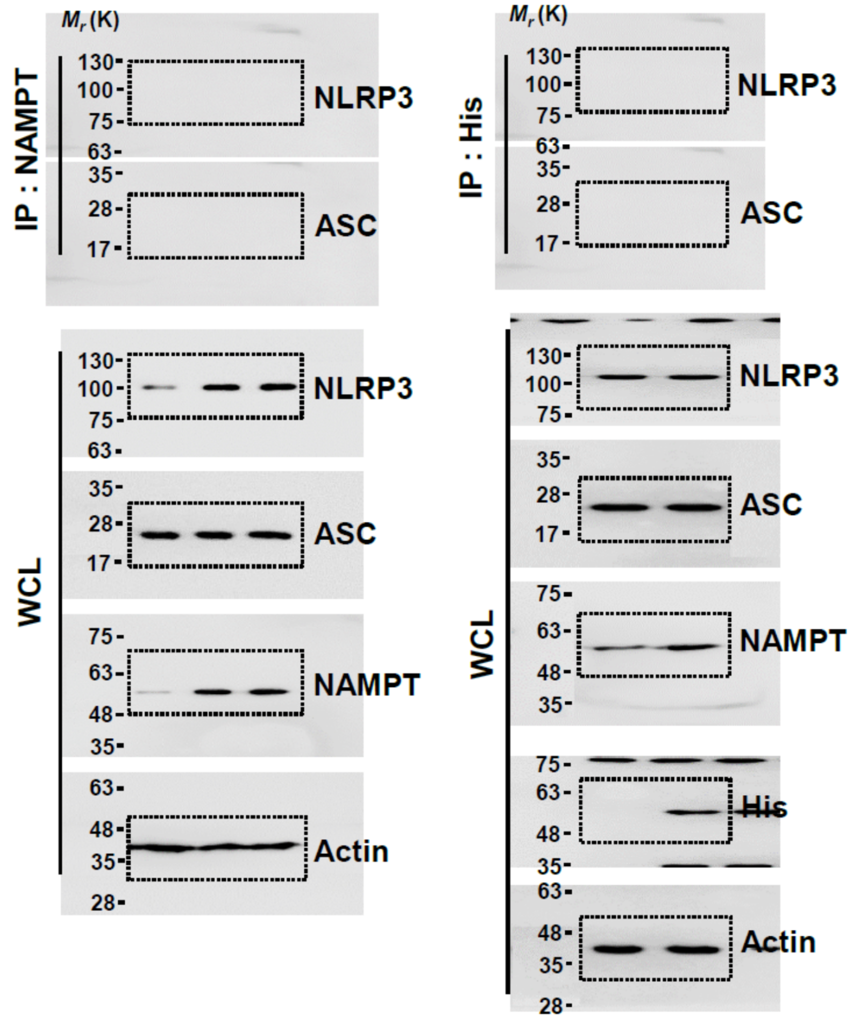

Fig 3A

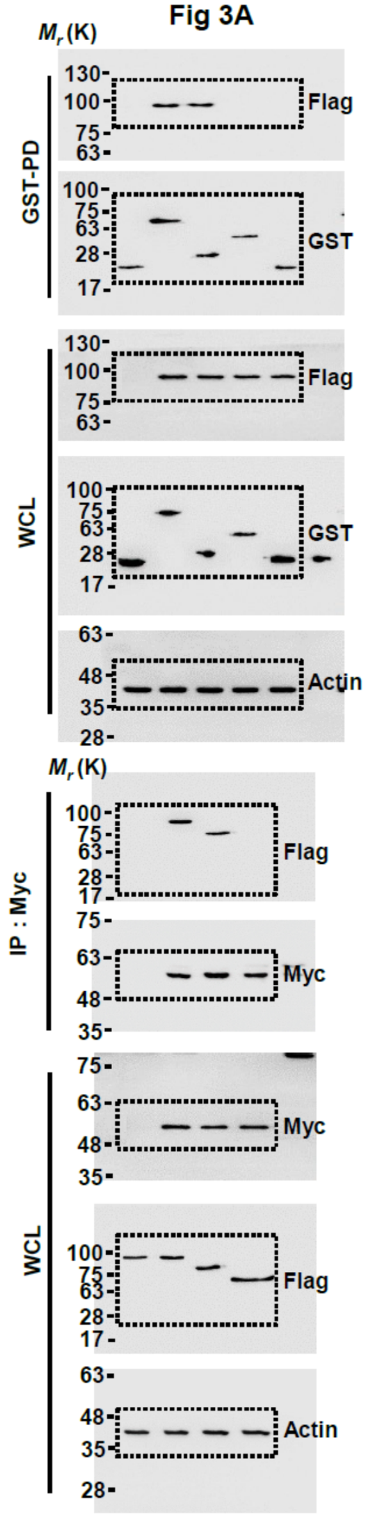

Fig 3B

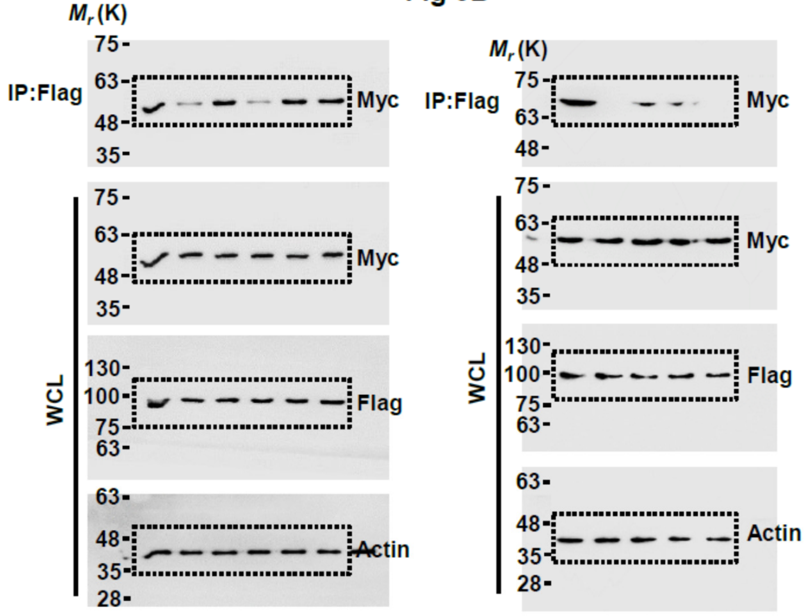

Fig 3C

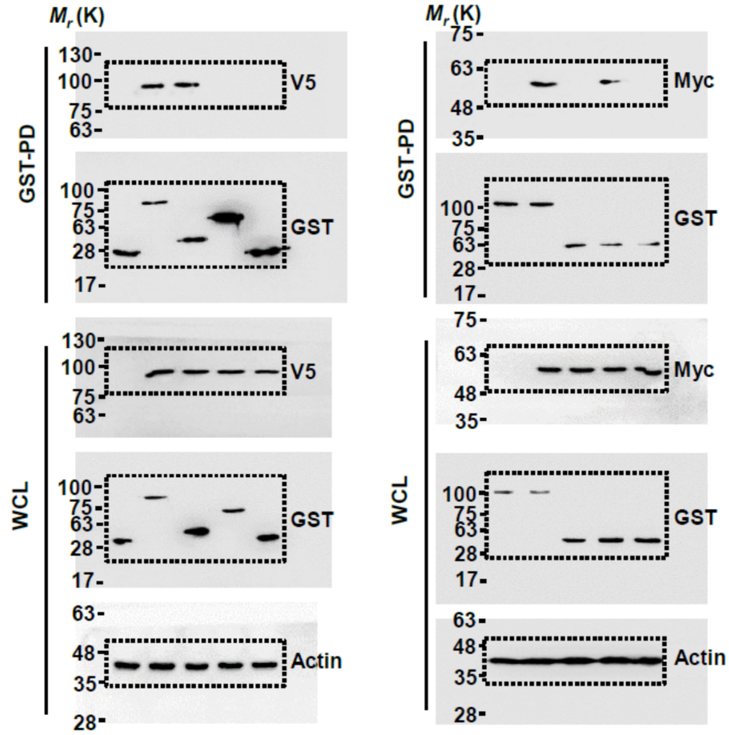

Fig 3D

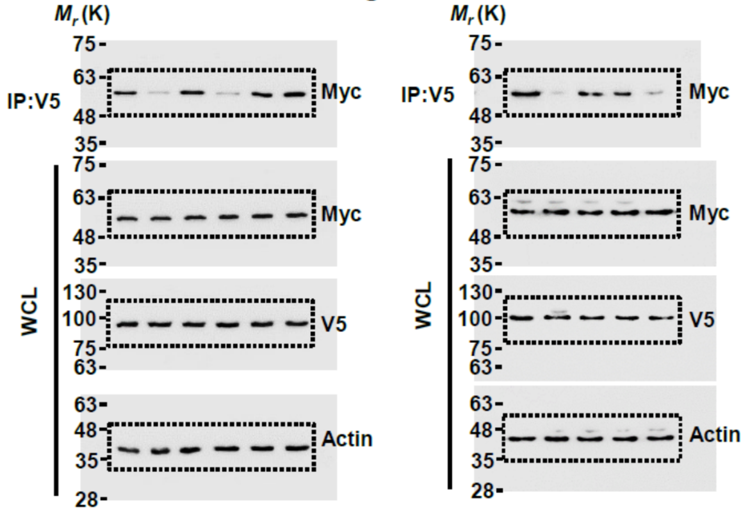

Fig 3E

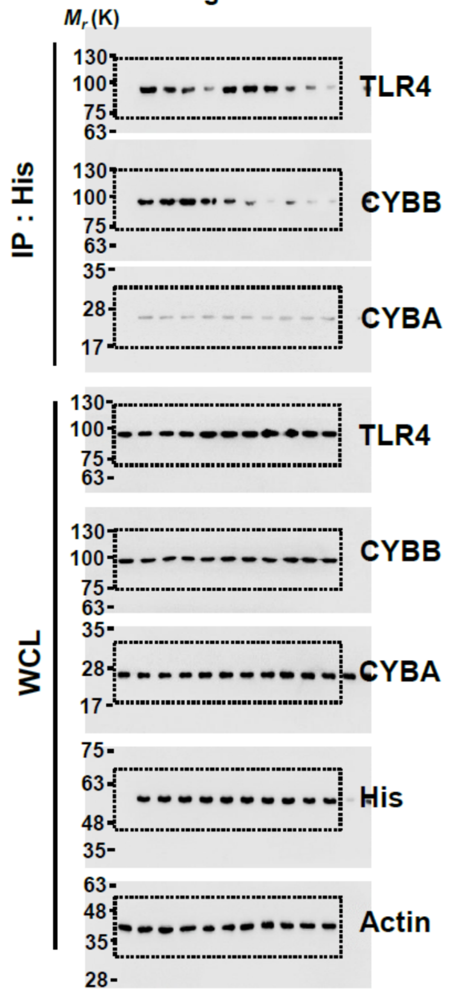

Fig 4A

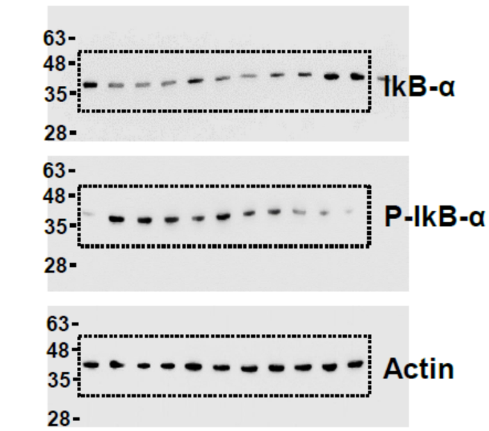

Fig 5A

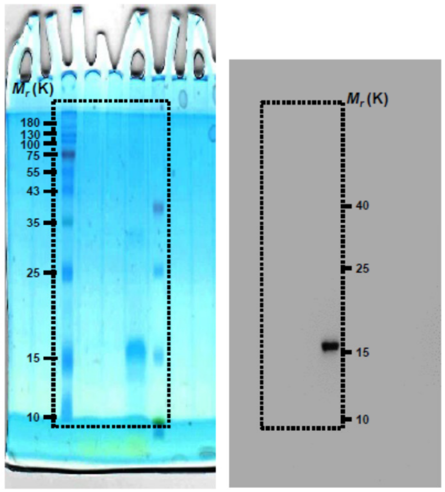

Fig 5B

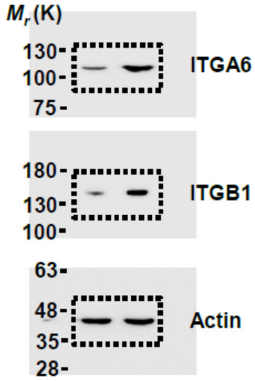

Fig 4E

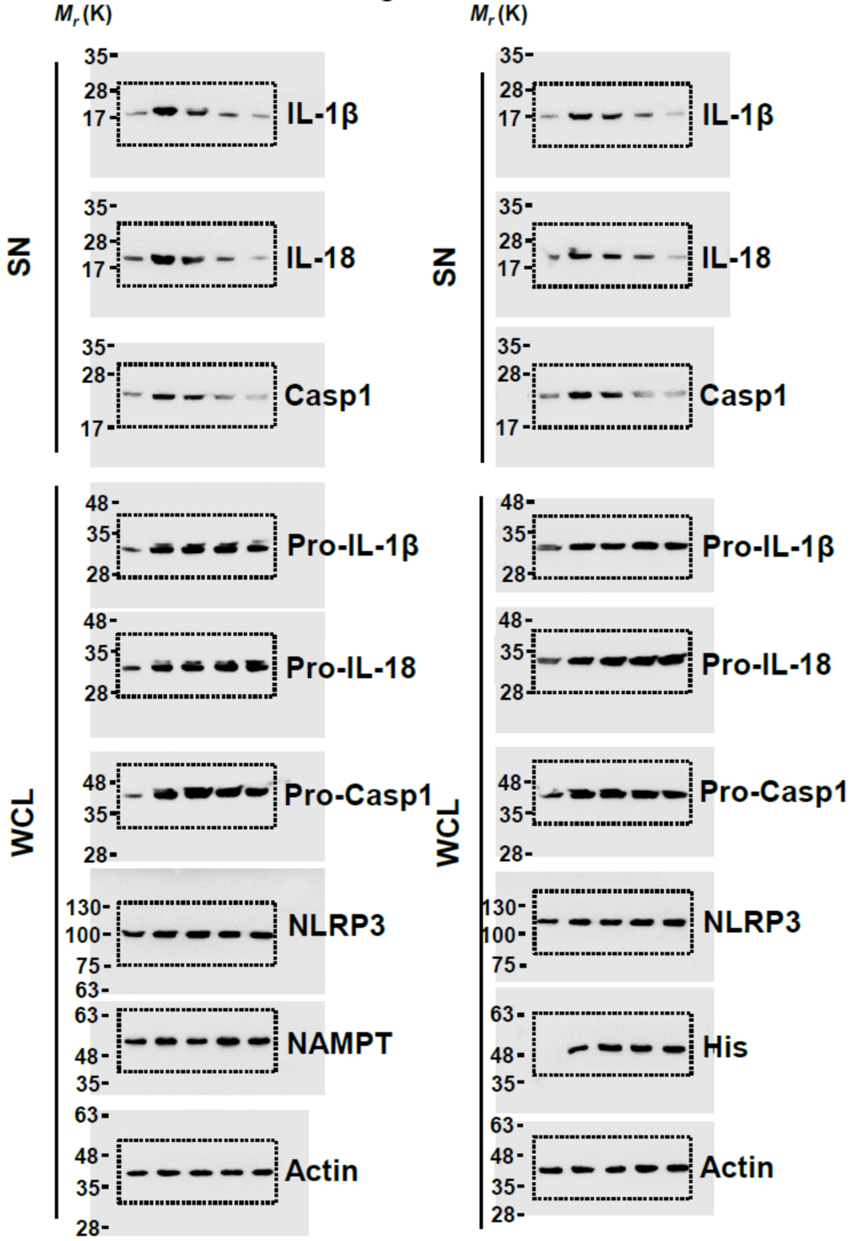

Fig 5C

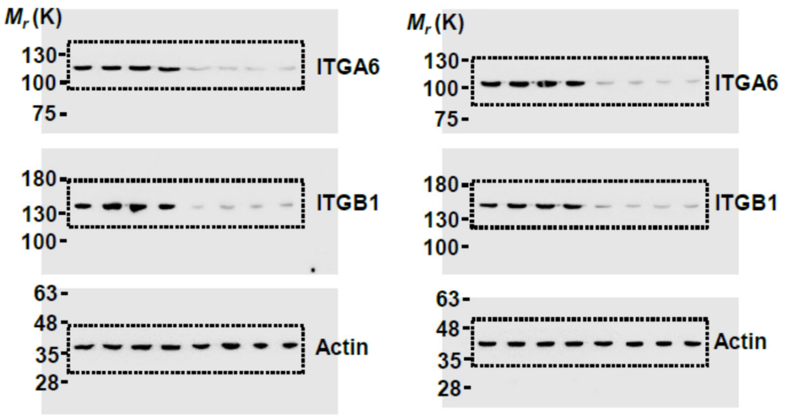

Fig 6E

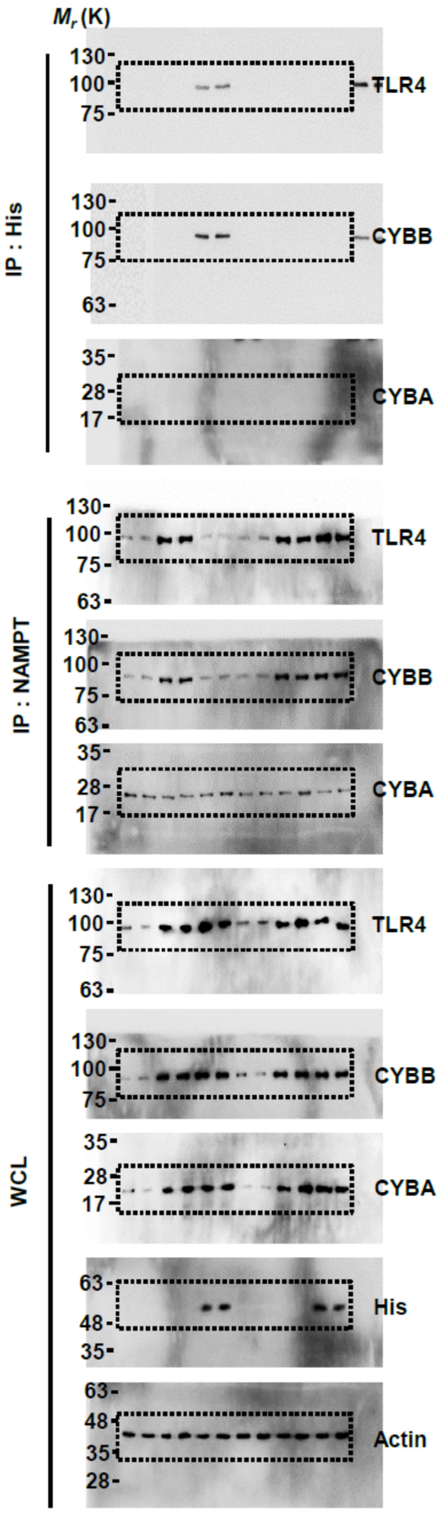

Table S1. A summary of gene set enrichment analysis for macrophage and T cells minor types. While macrophages have positive enrichment scores for many of inflammatory activities, such as interleukin signaling, innate immune system, cytokine/TNFα signaling and Toll-like receptor cascade, 3 T cell minor types showed mostly negative enrichment scores, except only for the "innate immune system" pathway in regulatory T cells, suggesting that T cells are in anti-inflammatory phase.

Cells	pathway	p.val	p.val.adj	NES
Macrophage	REACTOME Neutrophil Degranulation	5.54E-06	0.000	2.347
	REACTOME Signaling by Interleukins	4.45E-05	0.001	2.270
	REACTOME Innate Immune System	2.57E-05	0.001	2.107
	REACTOME Toll Like Receptor Cascades	0.003346	0.012	1.955
	HALLMARK Tnfa Signaling via Nfkb	0.002235	0.009	1.929
	HALLMARK Inflammatory Response	0.021467	0.062	1.756
	REACTOME Cytokine Signaling in Immune System	0.006648	0.022	1.728
	REACTOME Interleukin 4 and Interleukin 13 Signaling	0.016487	0.052	1.725
	WP Vitamin D Receptor Pathway	0.021583	0.062	1.607
	HALLMARK Complement	0.024264	0.066	1.583
	Class I MHC Mediated Antigen Processing Presentation	0.04918	0.113	1.545
	KEGG Leishmania Infection	0.030507	0.076	-1.630
	REACTOME Interferon Gamma Signaling	0.028175	0.073	-1.698
	WP Ebola Virus Pathway on Host	0.003064	0.011	-2.020
	KEGG Viral Myocarditis	0.001222	0.006	-2.038
	KEGG Intestinal Immune Network for Iga Production	0.001302	0.006	-2.076
	KEGG Asthma	0.000962	0.005	-2.125
	KEGG Allograft Rejection	0.000921	0.005	-2.127
	KEGG Autoimmune Thyroid Disease	0.000921	0.005	-2.127
	KEGG Graft Versus Host Disease	0.000921	0.005	-2.127
	KEGG Type I Diabetes Mellitus	0.000921	0.005	-2.127
	KEGG Antigen Processing and Presentation	0.000349	0.003	-2.187
	REACTOME Mhc Class Ii Antigen Presentation	0.000368	0.003	-2.188
	KEGG Systemic Lupus Erythematosus	5.97E-05	0.001	-2.313
	WP Allograft Rejection	4.28E-05	0.001	-2.333
Cytotoxic T cell	HALLMARK Interferon Gamma Response	0.022948	0.172	-1.751
	REACTOME Signaling by Gpcr	0.002779	0.041	-1.896
	HALLMARK Il2 Stat5 Signaling	0.002974	0.041	-1.963
	HALLMARK Tnfa Signaling via Nfkb	0.004098	0.041	-1.969
CD4 T cells	HALLMARK Allograft Rejection	0.037225	0.181	-1.583
	HALLMARK Apoptosis	0.012435	0.069	-1.856
	WP Il18 Signaling Pathway	0.011749	0.069	-1.868
	REACTOME Neutrophil Degranulation	0.005372	0.066	-1.874
	HALLMARK Interferon Gamma Response	0.008441	0.066	-1.895
	REACTOME Signaling by Gpcr	0.007646	0.066	-1.910
	HALLMARK Inflammatory Response	0.000406	0.008	-2.188
	HALLMARK Tnfa Signaling via Nfkb	1.28E-06	0.000	-2.707
Regulatory T cell	REACTOME Signaling by Gpcr	0.009243	0.025	-1.858
	HALLMARK Hypoxia	0.00407	0.016	-1.958
	HALLMARK Tnfa Signaling via Nfkb	0.000606	0.005	-2.180

* p.val: p-value, p.val.adj: adjusted p-value
